# The complete chloroplast genome sequence of *Dianyuea turbinata* (H.J. Dong & H. Peng) C. Shang, S. Liao & Z.X. Zhang (Salicaceae)

**DOI:** 10.1080/23802359.2021.1944362

**Published:** 2021-07-06

**Authors:** Dong Liu, Shenjian Xu, Feiyi Guo, Xuejiao Zhang, Zhiqi Yang, Ce Shang, Zhixiang Zhang

**Affiliations:** a School of Ecology and Nature Conservation, Beijing Forestry University, Beijing, China; b Museum of Beijing Forestry University, Beijing Forestry University, Beijing, China

**Keywords:** Chloroplast genome, phylogeny, Salicaceae, *Dianyuea turbinata*

## Abstract

The chloroplast genome sequences of *Dianyuea turbinata* (H.J. Dong & H. Peng) C. Shang, S. Liao & Z.X. Zhang, endemic to Yingjiang County, Yunnan Province, China, was presented in this study (Dong and Peng 2013; Shang et al. 2017). The chloroplast genome sequence was 154,045 bp in length, with a large single-copy (LSC) region of 82,247 bp, a small single-copy (SSC) region of 16,522 bp, separated by two inverted repeat (IR) regions of 27,638 bp each. The total GC content was 37.1%. The complete plastome sequence contained 134 genes, including 89 protein-coding, 37 tRNA and 8 rRNA genes. The phylogenetic status of genus *Dianyuea* has been clarified through a Maximum-likelihood tree based on the chloroplast genome of 17 species.

*Dianyuea* is a monotypic genus and belongs to Scyphostegioideae, which consists of only two species, *Dianyuea turbinata* and *Scyphostegia borneensis* Stapf. Species *Scyphostegia borneensis* is endemic to the northern part of Borneo, while *D. turbinata* was found from West Yunnan, China.

*Dianyuea turbinata* is a dioecious deciduous shrub. It could be easily recognized by thorns from leaf axils, monochlamydeous flowers with connate anthers or upper ovary with basal placenta, and seeds surrounded by fleshy appendages.

The fresh young leaves of *D. turbinata* were collected from Tongbiguan village, Yingjiang county, Yunnan, China (97.5871°E, 24.6067°N). Voucher specimens were deposited in the Herbarium of Beijing Forestry University (BJFC) (under collection numbers of *LD118*, Dong Liu, liudong0707@bjfu.edu.cn). Genomic DNAs were extracted using a genomic DNA extraction kit (Tiangen Biotech Co. Ltd., Beijing), and 2 × 150 bp pair-end sequencing was performed on an Illumina HiSeq 4000 platform at Novogene (http://www.novogene.com, China). We used Map to reference function of Geneious Primer 2020 (Kearse et al. 2012) to select chloroplast reads using published plastome sequence of *Salix* and *Populus* as reference (Chen et al. 2019). Then, these chloroplast reads were *de novo* assembled with Geneious Primer 2020. Gaps were filled using Fine Tuning function of Geneious Primer 2020. The assembled chloroplast sequence was annotated using the Plastid Genome Annotator (PGA, Qu et al. 2019), and verified by Geneious Primer 2020.

The complete chloroplast genome sequence of *D. turbinata* is 154,045 bp in length, with a large single-copy (LSC) region of 82,247 bp, a small single-copy (SSC) region of 16,522 bp, and two inverted repeats (IR) of 27,638 bp. The plastome contains 134 genes, including 89 protein-coding genes, 37 tRNA genes, and 8 rRNA genes. The total sequence GC content is 37.1%.

To clarify its phylogenetic position in the Salicaceae, a maximum-likelihood (ML) tree based on complete chloroplast genome sequences of 16 Salicaceae species with *Passiflora foetida* as outgroup from NCBI was reconstructed in PhyloSuite 1.1.16 (Zhang et al. 2020). All the sequences were aligned by MAFFT (Katoh et al. 2002). Alignment has 17 sequences with 188,364 columns, 155,644 of which are constant sites, 20,520 sites are singleton, and 12,200 sites are parsimony-informative. As shown in Figure 1, our research supports the fact that the genus *Dianyuea* is sister to the clade of the genera *Populus, Idesia, Flacourtia, Bennettiodendron, Carrierea, Passiflora, Xylosma, Homalium, Casearia, Itoa, Salix and Poliothyrsis*. Meanwhile, our study further clarifies the phylogenetic relationship among Salicaceae.

**Figure 1.**
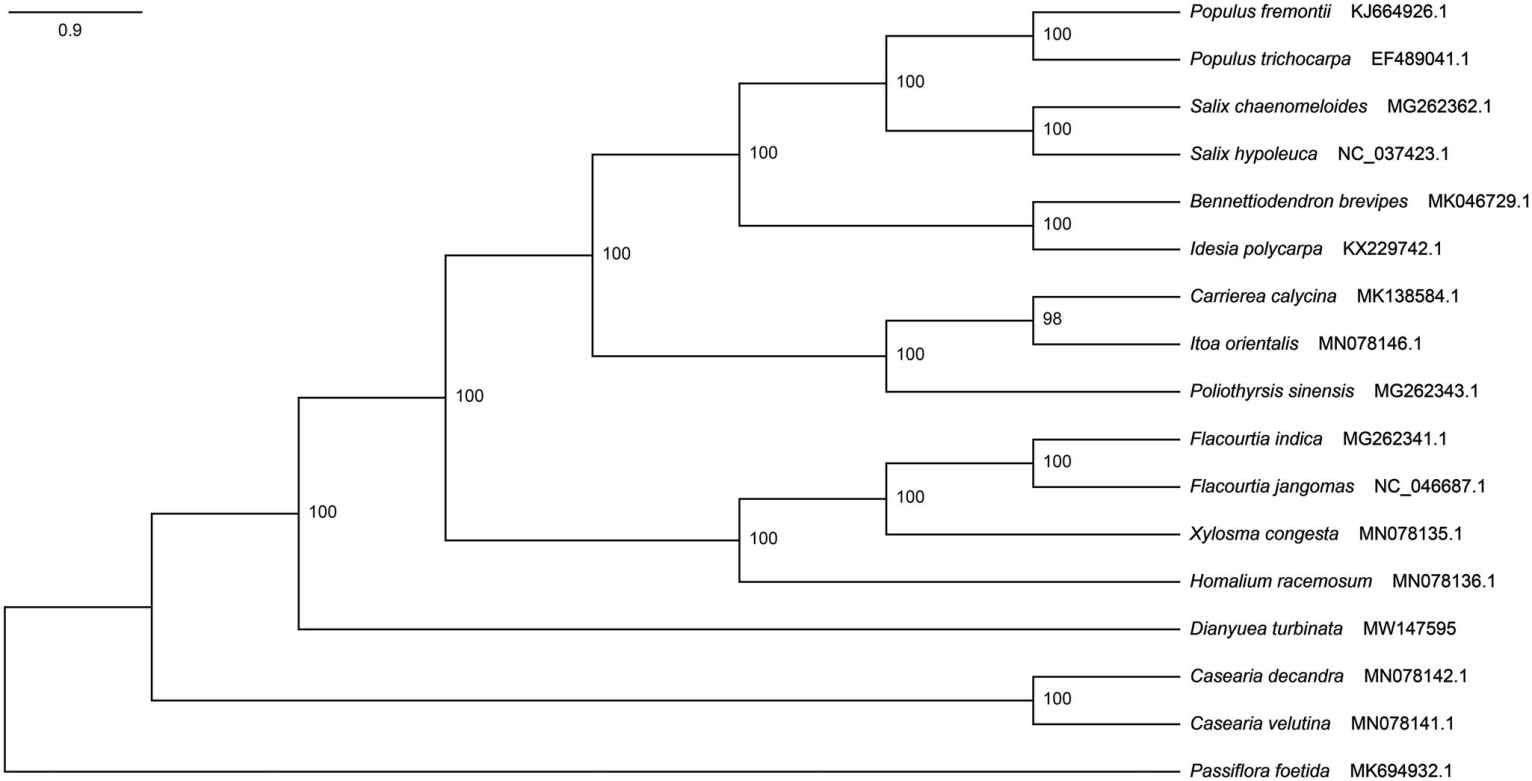
Maximum-likelihood phylogram of 16 Salicaceae species was reconstructed based on complete chloroplast genome sequences using Chen *Passiflora foetida* as an outgroup.

## Data Availability

The genome sequence data that support the findings of this study are openly available in GenBank of NCBI at [https://www.ncbi.nlm.nih.gov] (https://www.ncbi.nlm.nih.gov/) under the accession MW147595. The associated BioProject, SRA, and Bio-Sample numbers are PRJNA670816, SRR12881261, and SAMN16521137 respectively.
